# Understanding Home Health Agencies' Perspectives Toward Telehealth Use Among Home Health Stakeholders in the Post–COVID-19 Era: Qualitative Interview Study

**DOI:** 10.2196/75861

**Published:** 2025-08-07

**Authors:** Daniel Spertus, Kiran Abraham-Aggarwal, Mark Aaron Unruh, Jiani Yu, Kathryn H Bowles, Michael P Thompson, Cisco Espinosa, Hye-Young Jung, Madeline R Sterling

**Affiliations:** 1 Department of Medicine, Weill Cornell Medicine, 420 East 70th Street, LH-357, New York, NY, 10021, United States, 1 6469625029; 2 ILR School, Cornell University, Ithaca, NY, United States; 3 Department of Population Health Sciences, Weill Cornell Medicine, New York, NY, United States; 4 School of Nursing, University of Pennsylvania, Philadelphia, PA, United States; 5 Center for Home Care Policy & Research, VNS Health, New York, NY, United States; 6 Department of Cardiac Surgery, University of Michigan Medical School, Ann Arbor, MI, United States

**Keywords:** home health care, home health agencies, policy, Medicare, G-codes, qualitative study, telehealth

## Abstract

**Background:**

In the United States, the COVID-19 pandemic accelerated the adoption of telehealth in home health care (HHC), but its sustainability remains uncertain. Despite telehealth’s potential benefits, including improved patient monitoring and expanded access, the lack of reimbursement and regulatory constraints may limit widespread adoption. Understanding how home health agencies (HHAs) perceive these challenges is critical for shaping future telehealth policy.

**Objective:**

To examine HHA stakeholders’ perspectives on the adoption, implementation, and sustainability of telehealth in the postpandemic era, with particular attention to operational benefits, financial and regulatory barriers, and the impact of new Centers for Medicare & Medicaid Services (CMS) billing codes (G-codes) for telehealth documentation.

**Methods:**

Qualitative study using semistructured interviews conducted between February and December 2024. The study followed the Practical Implementation Sustainability Model (PRISM) framework for data collection and analysis. Participants were recruited from HHAs and home health policy organizations across the United States, representing a range of agency types and geographic regions. A purposive and snowball sampling strategy was used to recruit 14 stakeholders, including HHA leaders, HHC clinicians, and policy experts. Interviews were transcribed and analyzed thematically using both deductive codes from the PRISM framework and inductive codes to capture emergent themes. Participants described their experiences with telehealth in HHC, including its operational feasibility, clinical utility, financial impact, and response to new CMS G-codes introduced in July 2023 for telehealth documentation. Primary topics of focus included stakeholders’ perceptions of telehealth’s benefits, barriers, and future viability in HHC.

**Results:**

Stakeholders identified 4 key themes: (1) telehealth offers operational efficiencies (eg, increased patient touchpoints and workforce support) and clinical benefits (eg, improved patient monitoring and potential reduction in rehospitalizations); (2) the lack of CMS reimbursement makes telehealth adoption financially unsustainable for many HHAs; (3) specific HHAs, particularly those integrated with health systems or serving high-risk patient populations, may derive sufficient benefits to continue telehealth use despite financial constraints; and (4) current regulatory policies, including new CMS G-codes, increase administrative burden without providing financial incentives and discouraging telehealth adoption.

**Conclusions:**

While stakeholders recognize the benefits of telehealth in HHC, financial and regulatory challenges pose substantial barriers to its sustainability. Policymakers must weigh the advantages of telehealth reimbursement and regulatory support against concerns about wasteful care.

## Introduction

In the United States, the COVID-19 pandemic catalyzed a rapid and widespread shift in health care delivery from traditional clinical settings and face-to-face encounters to telehealth, which includes telephone calls, video visits, and remote patient monitoring (RPM) systems [1]. Initially, telehealth use was driven by the need to protect both providers and patients from COVID-19 and, in the United States, was facilitated by the Secretary of Health and Human Services using Section 1135 of the Social Security Act to temporarily modify certain Medicare, Medicaid, and Health Insurance Portability and Accountability Act (HIPAA) requirements [2,3]. These modifications allowed telehealth visits to be reimbursed at rates equivalent to in-person visits under fee-for-service payment models. In addition to being financially viable, observational cohort studies found telehealth to be associated with equivalent or improved outcomes (patient satisfaction, subsequent acute care usage, and mortality) across numerous patient groups, including older adults with chronic conditions, within primary care, subspecialties, and some long-term care settings [4-8]. Telehealth use in ambulatory care settings remains prevalent; a survey of 1,180,248 adults in the United States found that 25% of Medicare fee-for-service patients had a telehealth appointment in 2023 [9].

Beyond office-based and hospital settings, patients receiving care in skilled nursing facilities (SNFs) and from home health agencies (HHAs) in the United States have also benefited from Centers for Medicare & Medicaid Services’ (CMS) telehealth waiver. Prior to the pandemic, telemedicine usage in SNFs was low, accounting for just 0.15% of all resident visits. However, in early 2020, telehealth use increased to 15% of skilled nursing visits and 37% of outpatient visits among SNF patients, largely because of the COVID-19 pandemic. By 2022, telemedicine use stabilized at approximately 2% for skilled nursing visits and 8% for outpatient visits for SNF patients [10]

For HHAs in the United States, the COVID-19 waivers relaxed certain HIPAA requirements, providing HHAs greater flexibility in the selection of technologies and services for telehealth. Under the waivers, HHAs were also able to record their use of telehealth with temporary billing codes and billing code modifiers with the caveat that telehealth be included in the patients’ care plan and not replace in-person visits [11]. While this did not allow agencies to bill independently for telehealth, it allowed agencies to use remote services as a bridge between visits and reduced the need for in-person touchpoints. Recent evidence suggests that these changes significantly improved the accessibility and operational feasibility of telehealth for HHAs and their patients [12].

Despite the increased viability of telehealth use among HHAs, limited data on its adoption, sustained use, and associated outcomes at both the patient and agency level exist. One reason for this is that no formal reporting mechanisms existed by which HHAs could administratively account for these services prior to the temporary billing codes used during the COVID-19 public health emergency. In July 2023, CMS introduced 3 nonreimbursable G-codes which HHAs could use to account for telehealth services; G0320 allows for video visits, G0321 allows for phone call check-ins, and G0322 allows for RPM [13]. However, whether HHAs use these G-codes in the post-COVID-19 era is unclear.

To address these gaps, we conducted a multistate qualitative study to explore the perspectives of stakeholders in HHC toward the use of telehealth, as well as their attitudes toward CMS’ new G-codes. Specifically, our study aimed to understand (1) the perspectives of HHAs toward telehealth use, (2) whether adoption of telehealth changed as a function of new administrative G-codes, including whether these codes influenced adoption decisions, and (3) what the future holds for telehealth in HHC industry, including challenges and opportunities.

## Methods

### Study Setting and Design

We conducted this multistate qualitative study from February 2024 to December 2024. Stakeholders in home health included HHA leaders and staff, clinicians, and home health policy experts. We used a purposive sampling approach to recruit a diverse set of participants by HHA location and geography (region and urban or rural location) and profit status (for-profit and nonprofit) in the United States. We used a combination of strategies to identify eligible participants. These included outreach to stakeholders known to the research team through pre-existing studies, referrals from coauthors and collaborators, and cold outreach to individuals identified through publicly available organizational websites and directories. Using a prewritten electronic script, the lead study investigator (DS) emailed participants about the study. If participants were interested and met eligibility criteria, they provided electronic consent. All participants were offered a $50 gift card for participating.

### Data Collection

The lead investigator (DS), trained in qualitative methods, conducted single 30-minute, one-on-one, Zoom video conference interviews using a semistructured topic guide informed by the Practical Implementation Sustainability Model (PRISM), which expands the Reach, Effectiveness, Adoption, Implementation, Maintenance (RE-AIM) framework [14]. The PRISM domains include organizational and policy-level constructs that make it well suited to assess a broad array of experiences after the introduction of a policy change or intervention within organizational contexts.

Interview questions (Multimedia Appendix 1) broadly focused on stakeholders’ perspectives toward telehealth generally, including benefits and challenges with its use, as well as their attitudes and use of new G-codes to account for telehealth modalities, introduced by CMS in July 2023. We also sought to understand how HHA characteristics may serve as barriers and facilitators to telehealth use.

### Data Analysis

Interviews were audio recorded and professionally transcribed. Data were organized using Dedoose software (SocioCultural Research Consultants, LLC) and analyzed thematically, guided by the PRISM framework. A priori codes were used from the framework with our team adding inductive codes to account for new or emergent concepts. This led to an iteratively developed codebook. More specifically, 2 researchers (DS and KA) independently reviewed the first 3 transcripts and coded them independently using PRISM, adding new codes when appropriate. They met with the senior author, MRS, to reconcile and consolidate codes with disagreements resolved through discussion. The codebook was then reapplied to the initial and subsequent transcripts. Through discussion, codes were then categorized into subthemes and themes. Interviews continued until thematic saturation was reached, defined as the point at which no new themes emerged [15]. Themes were then reviewed with experts in home health (KB) and telehealth (JY) to support the trustworthiness of the findings. The research team brings backgrounds in medicine, public health, health policy, and qualitative research, which informed the study design and analysis. Team members’ professional experiences in home health care may have shaped the interpretation of findings; reflexive discussions and regular team debriefs were used to mitigate potential bias. To enhance rigor, we conducted dual coding of transcripts, developed the codebook iteratively, and continued interviews until thematic saturation was reached.

### Ethical Considerations

This study was approved by the Biomedical Research Alliance of New York (BRANY), the Institutional Review Board (IRB) of record (IRB #22-12-249-380), and acknowledged by the Weill Cornell Medical College IRB (IRB #22‐03024603). All participants provided electronic informed consent using an IRB-approved consent form. Interviews were audio recorded, professionally transcribed, and deidentified. Transcripts contained no identifying information and data were stored on secure, encrypted institutional servers. Participants were offered a $50 gift card by mail as compensation. This study adheres to the Consolidated Criteria for Reporting Qualitative Research (COREQ) reporting guideline (Checklist 1) as well as the Standards for Reporting Qualitative Research (SRQR).

## Results

### Baseline Characteristics

After reaching out directly to 41 stakeholders, 14 participants were interviewed (Table 1); participant roles ranged from clinical to operational. Four participants out of 14 participants (29%) were employed at for-profit HHAs, while 7 (50%) worked at nonprofit HHAs; 3 of the 7 nonprofit HHA participants were employed by HHAs integrated with a hospital/health system. Three participants (21%) were employed by home health–related policy and/or advocacy organizations. With respect to geographic location, 5 out of 14 participants (36%) represented groups from the Northeast and Mid-Atlantic regions of the United States, 2 from the Midwest (14%), and 2 from the West (14%). Five represented groups with a national scope (36%) (Table 1).

The thematic analysis resulted in 4 key themes, which are presented below with representative quotes (Textbox 1).

**Table 1.** Participant characteristics.

Characteristics	Total
Sex	
Female	11
Male	3
Home health agency type	
Free-standing	8
Integrated with hospital system	3
Home health agency profit status	
Nonprofit	7
For-profit	4
Stakeholder type	
Leaders within home health agency	6
Leaders within home health-related organizations	2
HHA clinician	3
Nonprofit (trade organization)	3
Region	
Midwest	2
Northeast/Mid-Atlantic	5
South	0
West	2
National	5
Rural	4

Textbox 1. **Key themes and subthemes.**

**Theme 1: Telehealth offers potential benefits.**

- Subthemes

-  Perceived benefits for HHAs
-  Perceived benefits for patient care

**Theme 2: Despite benefits, the lack of reimbursement for telehealth is problematic.**

- Subthemes

-  Financially prohibitive for HHAs
-  Administrative costs associated with telehealth use
-  Exacerbates general Financial Hardship for HHAs

**Theme 3: While widespread adoption of telehealth appears unlikely, certain HHAs and patient populations may benefit.**

- Subthemes

-  Distinct patient populations for use
-  HHA characteristics that support telehealth

**Theme 4: Without changes to policy and payment structures, the use of telehealth in home health appears generally unsustainable.**

- Subthemes

-  COVID-19 regulatory waivers facilitated telehealth use in HHC
-  The return to prepandemic regulations and increased reporting requirements disincentivize HHA telehealth use
-  HHAs believe that lack of reimbursement for telehealth in HHC is driven by CMS’ mistrust of agencies

### Theme 1: Telehealth Offers Potential Benefits for Both HHAs and Patients

Participants consistently emphasized the benefits of telehealth, not only for their HHAs but also for the patients they serve. Participants discussed telehealth’s operational benefits in HHAs, including its capacity to support clinicians in seeing more patients. Two participants said:

*If you want to maximize how many patients you’re touching, being able to balance what could be done through either a phone call or a video call versus in person can help you be able to see more patients and have more oversight into how they’re doing*.

*We can see about double the patients virtually in a day per clinician than going out into the field.*

Others noted how telehealth can help them overcome capacity challenges, particularly given the rise in HHA workforce shortages during the COVID-19 pandemic. A participant remarked,

*[Telehealth] does potentially allow you to touch more patients than you would traveling vast geographies to try and see patients*.

Beyond benefits to HHA workflow and operations, participants highlighted the clinical benefits of telehealth services, particularly RPM. Telehealth technology allows HHAs to enhance day-to-day symptom monitoring and, in turn, clinical management decisions. As 1 participant explained,

*[We are] more in tune with what’s happening with them on a day-to-day basis… we now know when there is an issue and can better address it.*

Given that patients of HHAs are particularly vulnerable to rehospitalizations, improvements in regular monitoring also have the potential to reduce the likelihood of these events. A participant said,

*Rehospitalizations went from like 1-in-4 to 1-in-20… the data was just concrete that [telehealth] was expertly effective in preventing folks from having to go back to the hospital.*

### Theme 2: Despite Benefits, the Lack of Reimbursement Is Problematic

Many participants reported that, despite telehealth’s benefits to both HHAs and patients, its implementation was and is significantly hindered due to services not being reimbursed. Without reimbursement, the costs associated with telehealth implementation for HHAs are difficult to overcome. One agency leader explained this by saying,

*I am pretty sure that if home health was given reimbursement for a video visit, that we would see more of it happening because it’s efficient and it can prevent a gap in care*.

A policy advocate participant stated,

*It’s not really discussed much amongst providers or at the meetings I attend, likely because there’s no current reimbursement*.

Participants also discussed how the administrative expenses associated with implementing and maintaining telehealth systems create additional barriers. As 1 participant noted,

*[Telehealth] investment really does require upfront capital and a lot of time working with staff to make sure that they understand*.

Another participant explained,

*As administrative requirements on this increase with CMS attempting to measure [telehealth use] without providing actual reimbursement, at some point, the scale is going to tip and we’re going to say this is too costly to even manage*.

Furthermore, several participants highlighted the need and cost for additional staff when telehealth is used:

*We have to have separate clinicians who are involved with reading the dashboards, identifying patients that need immediate help, and then coordinating with field staff. These are all components that aren’t paid for*.

Another participant captured the widespread feeling of financial strain facing the home health community that further inhibits spending on nonreimbursed services by stating,

*There’s also just the general sense from the broader home health community that the payment system right now is being cut consistently*.

The cumulative burden of these implementation and staffing costs, coupled with the lack of reimbursement, may make the provision of telehealth unsustainable for HHAs, which already face financial challenges.

### Theme 3: While Widespread Adoption of Telehealth Appears Unlikely, Certain HHAs and Patient Populations May Benefit

Despite financial barriers limiting the widespread adoption of telehealth at HHAs, participants spoke about certain contexts in which telehealth may offer value. For example, specific patient populations with high-risk conditions, such as those with heart failure, were mentioned as populations that would benefit more than others from telehealth services.

*We’ve been focused on patients with congestive heart failure for the most part. We had a broader population in the past, but we found it to be most useful in preventing hospitalization with that group*.

In addition, participants spoke about certain HHA characteristics such as being in rural areas or being integrated with a health system that made using telehealth more appealing and/or more feasible. One participant explained that smaller, stand-alone agencies may struggle with the finances of offering telehealth, whereas larger, more profitable ones might be able to make this work.

*I think larger agencies can afford it, at least they can dabble a little bit and figure out what’s going to work. A small agency that is not bringing in a huge revenue stream is just going to see this as another cost*.

Rural HHAs, in particular, may stand to benefit from telehealth according to some of the participants. This is because the service may allow them to overcome large physical distances to provide services, as 1 participant explained:

*I definitely think telehealth is a great benefit to rural type of home care agencies. In some cases, they have even more vast geographies than some of the metropolitan areas, and they don’t have as many resources*.

### Theme 4: Current Regulatory and Billing Policies Do Not Facilitate Telehealth Use in HHAs

While telehealth saw increased usage during the COVID-19 pandemic due to temporary regulatory flexibilities, the return to prepandemic regulations has introduced new challenges that compound other financial barriers.

*Now that the public health emergency is over, [HHAs] have to use HIPAA-compliant technology, which is more expensive*.

One agency leader summarized the industry’s frustration with CMS’ post–COVID-19 telehealth policy for HHC by asserting,

*CMS created perfect disincentives to do it. Right. [They] made reporting requirements while saying that these visits and services cannot count towards home health payment.*

When asked about the future of telehealth reimbursement, participants often expressed the belief that policymakers and regulators, wary of potential fraud or abuse, approach telehealth payment for HHAs with caution. As 1 participant succinctly explained,

*There is a lot of fear, both from regulators and Congress, on abuse of [telehealth-based HHC]*

## Discussion

### Principal Results

This qualitative study explored the perspectives of stakeholders in HHC toward telehealth, including operational and clinical benefits, responses to new administrative G-codes, and what the future may hold for these services. Our participants shared a clear belief in telehealth’s ability to enhance care delivery, efficiency, and outcomes, particularly in resource-constrained settings. However, the lack of reimbursement for telehealth services provided by HHAs impedes its widespread adoption. Stakeholders also expressed frustration over the regulatory challenges and administrative burdens associated with implementing telehealth, including the new G-codes. Despite these challenges, integrated and rural HHAs and specific patient populations, like those with heart failure, may continue to benefit from telehealth, even without changes to current policies.

A key finding was the tension between potential benefits and current billing and regulatory policies. While nearly every participant expressed a strong belief that telehealth could benefit their agency’s workflow or patient care, they also stated that it is not financially viable for most HHAs. Our findings suggest this is likely due to widespread financial challenges in the industry which are compounded by CMS’ decision to require reporting of telehealth use without providing reimbursement.

Somewhat paradoxically, our participants also believed that cost-effective telehealth could alleviate some of the financial pressures in the industry, particularly as HHAs face a severe workforce shortage—a trend that is projected to worsen in the coming years [16,17]. However, navigating complex and seemingly ever-changing reimbursement systems while managing staffing shortages has left agencies financially strained and with little opportunity to invest in the infrastructure, technology, and training required for telehealth adoption.

Stakeholders in our study expressed tempered optimism about the short-term future of telehealth reimbursement in HHC. Many participants shared a belief that CMS is suspicious of HHAs and the broader HHC industry, frequently noting that CMS seems to impose excessively restrictive regulations on the industry, apparently driven by concerns over waste and abuse. Our participants felt that the current telehealth reimbursement policy exemplified this stance. However, from CMS’ perspective, the central debate over telehealth reimbursement hinges on whether it provides meaningful value [18]. If implemented ineffectively, telehealth has the potential to contribute to unnecessary health care usage. For instance, a telemedicine visit that ultimately necessitates an in-person follow-up—when the issue could have been resolved in a single in-person visit—creates a risk of redundant payments by CMS without improving clinical outcomes. Our participants acknowledged that many home health services inherently necessitate the physical presence of a health care professional to provide hands-on care and assess home environments for safety risks.

There may, however, still be some viable applications for telehealth technologies in HHC without direct reimbursement. A second key finding was that rural and integrated HHAs and high-risk patient populations may derive sufficient benefits from telehealth to make its use financially sustainable for HHAs. For rural HHAs, telehealth can help expand census by reducing the need for in-person touchpoints. Integrated HHAs may also benefit from telehealth by building on preexisting infrastructure (eg, hospital-based RPM that could continue into a home health episode). For high-risk patient populations, including those with chronic conditions such as heart failure, where symptom exacerbations can be monitored quickly with virtual touchpoints and RPM, telehealth may reduce hospitalizations; this may be particularly beneficial for HHAs participating in value-based purchasing reimbursement models [19]. However, the use and benefit of telehealth among these HHA agencies and patients need to be examined in observational and trial-based studies [20]. Beyond getting a broader sense of telehealth effectiveness, future work should also aim to understand what home care situations are most amenable to telehealth and if telehealth is likely to replace or add to overall home care use. Based on our findings, we recommend that CMS consider better aligning telehealth documentation requirements with reimbursement policies. For example, modest reimbursement could be tied to the use of G-codes to reduce administrative burden and incentivize uptake. Policymakers might also explore pilot reimbursement programs targeting high-need populations, such as those with high clinical risk or in rural areas, or agencies already demonstrating operational efficiencies from telehealth use.

### Limitations

This study has several limitations. First, while we aimed to include HHAs and participants diverse in regional and payer characteristics, our sample favors certain perspectives, namely those from stakeholders associated with nonprofit HHAs in the Northeastern United States. While we reached thematic saturation, our findings cannot be transferred to regions or agency types that were not included. Second, while we included many stakeholder types, we did not include telehealth recipients, namely patients and their family caregivers, and thus lack their perspectives. Third, our sample lacked representation from the Southern United States, a region with distinct health care delivery structures and a large rural population that could particularly benefit from telehealth. Finally, because our study was conducted entirely in the United States, its findings may not translate to other health care systems.

### Comparison With Prior Work

Since the onset of the pandemic, research examining the use of telehealth in ambulatory care settings has broadly demonstrated comparable or improved outcomes when compared to traditional, in-person care [4-8]. However, evidence demonstrating post-COVID trends in the usage of these services and their associated outcomes in home health is lacking. Although some pre-2020 research identified trends in home health telehealth use in the United States and its effectiveness in specific contexts, the rapidly evolving landscape since their publication has rendered much of that information outdated [21-25]. Our study begins to address this gap by identifying stakeholder perspectives toward HHA telehealth and what it would take for its use to continue.

### Conclusions

Our findings suggest that while telehealth has perceived benefits for HHAs and patients in this setting, financial barriers have dampened its implementation. The recently introduced G-codes, meant to encourage telehealth use by allowing for documentation, may have instead imposed additional administrative burdens on agencies that are already dealing with thin financial margins. While participants remained optimistic about the role of telehealth in HHC, especially among rural and integrated agencies and certain patient subgroups, this optimism was moderated by the lack of reimbursement and skepticism that future policies will benefit the industry.

## Supplementary material

10.2196/75861 Multimedia Appendix 1 Telehealth topic guide.

10.2196/75861 Checklist 1 COREQ Checklist.

## Abbreviations

CMS: Centers for Medicare & Medicaid Services
COREQ: Consolidated Criteria for Reporting Qualitative Research
HHA: home health agency
HIPAA: Health Insurance Portability and Accountability Act
IRB: Institutional Review Board
PRISM: Practical, Robust Implementation and Sustainability Model
RE-AIM: Reach, Effectiveness, Adoption, Implementation, Maintenance
RPM: remote patient monitoring
SNF: skilled nursing facility
SRQR: Standards for Reporting Qualitative Research
