# Supplementary material for: Understanding Home Health Agencies' Perspectives Toward Telehealth Use Among Home Health Stakeholders in the Post–COVID-19 Era: Qualitative Interview Study
Source: J Med Internet Res. 2025 Aug 7;27:e75861. doi: 10.2196/75861 (PMC12336361; doi:10.2196/75861)
Supplement: Multimedia Appendix 1 [file jmir-v27-e75861-s001.docx]

*Interview Guide: Home Health Telehealth Usage*

**Interview Date:**

**Interviewer:**

**Interviewee:**

**Intro:**

- Since COVID, more providers are using telehealth. Over the summer, Medicare released three new billing codes to allow home health agencies to report their use of telehealth. The new home health billing codes cover the following three uses for telehealth: Video visits: “Home health services furnished using synchronous telemedicine rendered via a real-time two-way audio and video telecommunications system”
- Phone call visits: “Home health services furnished using synchronous telemedicine rendered via telephone or other real-time interactive audio-only telecommunications system”
- Remote monitoring: “The collection of physiologic data digitally stored and/or transmitted by the patient to the home health agency (for example, remote patient monitoring)”

**Questions:**

1. Does your agency provide any of these telehealth services?
   1. If yes:

Phone calls and video visits

- - 1. Which?

RPM, phone calls, started during the pandemic

- - 1. Who from your agency is doing this?
       1. Do you have dedicated telehealth staff?
    2. In what circumstances are you using telehealth and why?
    3. What are the pros of conducting telehealth visits in home health?
    4. What are the cons of conducting telehealth visits in home health?
  1. If no, why not?
     1. What are some of the barriers that may exist to providing these services?

1. The new billing codes became available for use on July 1^st^ of 2023. Is your agency using them for reimbursement?
   1. If they are:
      1. How has the process of adopting these been?
         1. Are there barriers or complications to using the codes?
   2. If they are not:
      1. Why not?
      2. Are there barriers or complications that make it impractical to utilize the codes?
2. Are there particular patients you are more likely to use telehealth for?
   1. Do you find that patients and caregivers like telehealth? Why or why not?
3. What are you hearing in general about adoption of telehealth and these billing codes in the home care industry?
   1. Are many agencies using them? Why or why not?
   2. Which agencies do you think are more likely to provide these services and/or use these codes?
      1. Size (large or small)
      2. Access to technology
      3. Urban/rural
4. Does your agency have a plan to expand or contract its use of telehealth in the future? Why? Why not?
5. Is there anything you want to add that we haven’t asked you?

Thank you for your time. We really appreciate it.
